# Epidemiology of systemic sclerosis: a multi-database population-based study in Tuscany (Italy)

**DOI:** 10.1186/s13023-021-01733-4

**Published:** 2021-02-17

**Authors:** Alessio Coi, Simone Barsotti, Michele Santoro, Fabio Almerigogna, Elena Bargagli, Marzia Caproni, Giacomo Emmi, Bruno Frediani, Serena Guiducci, Marco Matucci Cerinic, Marta Mosca, Paola Parronchi, Renato Prediletto, Enrico Selvi, Gabriele Simonini, Antonio Gaetano Tavoni, the Rare Diseases Working Group, Fabrizio Bianchi, Anna Pierini

**Affiliations:** 1 Institute of Clinical Physiology, National Research Council, Via Moruzzi 1, Pisa, Italy; 2 Rheumatology Unit, Department of Clinical and Experimental Medicine, University of Pisa, Pisa, Italy; 3 Immunoallergology Unit, , Careggi University Hospital, Florence, Italy; 4 Respiratory Diseases and Lung Transplantation, Department of Medical and Surgical Sciences and Neurosciences, University of Siena, Siena, Italy; 5 Rare Dermatological Diseases Unit, USL Toscana Centro, Firenze, Italy; 6 ERN-SKIN Diseases Centre, Department of Health Sciences, University of Florence, Firenze, Italy; 7 Department of Experimental and Clinical Medicine, University of Firenze, Firenze, Italy; 8 Rheumatology Unit, Department of Medical Sciences, Surgery and Neurosciences, University of Siena, “Le Scotte” Hospital, Siena, Italy; 9 Rheumatology Unit, Department of Clinical and Experimental Medicine, University of Florence, Florence, Italy; 10 Fondazione Toscana “Gabriele Monasterio”, Pisa, Italy; 11 Rheumatology Unit, Azienda Ospedaliero Universitaria Senese, Siena, Italy; 12 Rheumatology Unit, A. Meyer Children’s University Hospital, University of Florence, Florence, Italy; 13 Clinical Immunology Unit, Department of Clinical and Experimental Medicine, University of Pisa, Pisa, Italy

**Keywords:** Systemic sclerosis, Survival, Mortality risk, Comorbidity, Disease registry, Rare disease, Pharmacoepidemiology

## Abstract

**Background:**

Systemic Sclerosis (SSc) is a chronic autoimmune disease with a complex pathogenesis that includes vascular injury, abnormal immune activation, and tissue fibrosis. We provided a complete epidemiological characterization of SSc in the Tuscany region (Italy), considering prevalence and incidence, survival, comorbidities and drug prescriptions, by using a multi-database population-based approach. Cases of SSc diagnosed between 1st January 2003 and 31st December 2017 among residents in Tuscany were collected from the population-based Rare Diseases Registry of Tuscany. All cases were linked to regional health and demographic databases to obtain information about vital statistics, principal causes of hospitalization, complications and comorbidities, and drug prescriptions.

**Results:**

The prevalence of SSc in Tuscany population resulted to be 22.2 per 100,000, with the highest prevalence observed for the cases aged ≥ 65 years (33.2 per 100,000, CI 95% 29.6–37.3). In females, SSc was predominant (86.7% on the total) with an overall sex ratio F/M of 6.5. Nevertheless, males presented a more severe disease, with a lower survival and significant differences in respiratory complications and metabolic comorbidities. Complications and comorbidities such as pulmonary involvement (HR = 1.66, CI 95% 1.17–2.35), congestive heart failure (HR = 2.76, CI 95% 1.80–4.25), subarachnoid and intracerebral haemorrhage (HR = 2.33, CI 95% 1.21–4.48) and malignant neoplasms (HR = 1.63, CI 95% 1.06–2.52), were significantly associated to a lower survival, also after adjustment for age, sex and other SSc-related complications. Disease-modifying antirheumatic drugs, endothelin receptor antagonists, and phosphodiesterase-5 inhibitors were the drugs with the more increasing prevalence of use in the 2008–2017 period.

**Conclusions:**

The multi-database approach is important in the investigation of rare diseases where it is often difficult to provide accurate epidemiological indicators. A population-based registry can be exploited in synergy with health databases, to provide evidence related to disease outcomes and therapies and to assess the burden of disease, relying on a large cohort of cases. Building an integrated archive of data from multiple databases linking a cohort of patients to their comorbidities, clinical outcomes and survival, is important both in terms of treatment and prevention.

## Background

Systemic Sclerosis (SSc) is a rare chronic autoimmune disease with a complex pathogenesis that includes vascular injury, abnormal immune activation, and tissue fibrosis [1]. The disease is typically characterized by skin fibrosis, however internal organs may also be frequently affected, in particular kidneys, heart, lungs, and the gastrointestinal tract, thus dramatically reducing the patient’s quality of life and survival [2].

Despite the recent advance in the treatment, the prognosis of the patients is still severe and a high percentage of patients mainly dies for pulmonary fibrosis, pulmonary arterial hypertension (PAH) and cardiac disease (mainly heart failure and arrhythmias) [3].

Given the complexity of the study of the epidemiology of SSc, the prevalence in Europe is heterogeneous, ranging from 10 to 35 cases per 100,000 inhabitants [4]. This great difference is mainly due to different time-frames, study designs, data sources (hospitals database, general practitioners, public health data), and classification criteria. Some environmental factors may also influence the prevalence of SSc that appears to be higher in South Europe [5]; furthermore, a geographical variation is observed for several clinical aspects [6].

Most of the published epidemiologic studies on SSc were based on cases collected from records of general practitioners and/or from hospital database [7–16] or from health databases selecting records with the International Classification of Diseases (ICD) code for SSc included in the discharge diagnoses [17, 18].

To our knowledge, no studies have been published on SSc based on population-based registries or surveillance registries that, by their nature, are characterized by a high degree of completeness of case ascertainment (external completeness).

Disease registries are considered powerful instruments to develop clinical research in the field of rare diseases (clinical-, hospital-based registries), to improve patient care, and to support healthcare planning through the production of epidemiological indicators based on a specific geographical area (public health-, population-based registries) [19].

Population-based registries often contain information from a large range of conditions, but usually lack of clinical data. This is in contrast with disease-specific registries, that focus on a single disease or on related groups of diseases and have more clinical background [20].

Rare diseases registries, both clinical and population-based, are an important tool to collect a critical mass of data for epidemiological and/or clinical research, contributing to understand the natural history of rare conditions and to constitute a key information system that supports the activities of the European Reference Networks (ERNs) on rare diseases.

An integrated multi-database, established through the linkage of a population-based registry and data available from health databases routinely collected at geographic level, such as hospital discharge records, prescription database, and vital status database, can be a powerful tool to provide evidence related to disease outcomes and therapies and to assess the burden of disease.

This study is aimed at providing a complete epidemiological profile of SSc in Tuscany (Italy), characterizing the study population in terms of survival, comorbidities and drug prescriptions, by using a multi-database population-based approach.

## Methods

Cases of SSc diagnosed between 1st January 2003 and 31st December 2017 and residing in Tuscany, an Italian region with a population of 3,744,398 inhabitants, were collected from the population-based Rare Diseases Registry of Tuscany [21]. The registry is based on a regional network allowing the detection of all cases diagnosed at any age, by any of the regional health centres and it is one of the main contributors to the Italian National Registry of Rare Diseases at the Italian National Institute of Health [22].

SSc was diagnosed according to the criteria proposed by the American Rheumatism Association in 1980 [23] and/or the European League Against Rheumatism collaborative initiative/American College of Rheumatology (EULAR/ACR) criteria [24].

All cases endowed of a unique regional anonymous identification number were linked to health and demographic databases of the Tuscany Region to obtain information about vital statistics, hospitalization, complications and drug prescriptions.

Incidence was calculated by 5-years periods and for the whole study period. Prevalence was calculated at 31st December 2017 (population of Tuscany: 3,736,968 inhabitants); information about deaths and migrations necessary to produce prevalence estimates were gained from regional health and demographic databases (mortality database, Registry Office, hospital discharges database). Population stratified by sex and age was extracted by the National Institute of Statistics.

A Poisson regression model was used for testing differences in prevalence among age classes (< 25, 25–44, 45–64, ≥ 65). Mean age at diagnosis was expressed in years ± standard deviation.

The most frequent SSc-related complications and comorbidities were investigated according to the recent literature [25, 26] and were defined using the hospital discharge database with the International Classification of Diseases, Ninth Revision, Clinical Modification (ICD-9-CM) diagnosis codes (see Additional file 1: Table S1).

Principal causes of hospitalization (calculated on the total of inpatient cases), Length of Stay (LOS), i.e. the number of nights the patient remained in the hospital, and average LOS were also provided. Sex differences in SSc-related complications/comorbidities and principal causes of hospitalization were tested using the test for the difference between proportions.

Survival for SSc-cases was defined with the first registration of diagnosis and censored at death or at 1, 3, 5, and 10 years follow-up, using the Kaplan–Meier method and survival curves were compared using the log-rank test. The analysis of the effect of SSc-related complications and comorbidities on survival, adjusted by age and sex, were performed using Cox proportional hazards regression.

Drug prescriptions database, containing information on dispensed drugs reimbursed by the National Health Service, was available for this study from 1st January 2008. Only outpatient prescriptions were collected in the database. The prevalence of use of the most common classes of prescribed drugs in SSc (see Additional file 1: Table S2) was calculated for each year of the 2008–2017 period, by dividing the number of SSc cases with at least one dispensing of each pharmaceutical class for the number of prevalent cases at the beginning of each year. Defined Daily Dose (DDD) per 1000 cases per day (DDD/1000 cases/day), was used as an estimate of the proportion of the study population daily treated with a particular drug or group of drugs: 10 DDDs per 1000 cases per day means that in a representative group of 1000 SSc cases, 10 DDDs of the drug are utilized on average, on any given day of the year analysed. Investigated drugs were selected according to EULAR and EUSTAR (EULAR Scleroderma Trials and Research group) recommendations and updates for the treatment of SSc and to literature [27–29]. The Anatomical Therapeutic Chemical (ATC) classification system was used to code drugs information.

The data were analysed with STATA, version 16 [30]. Two-sided *p*-value < 0.05 was considered statistically significant in all analyses of this study.

Prior to their insert in the Rare Diseases Registry of Tuscany, an informative note, specifying that data can be used for research purpose, is delivered to each patient.

## Results

A total of 924 cases of SSc (801 females and 123 males) were diagnosed during the 2003–2017 period. Distribution of cases by 10-years age-classes was as follows: 0–17: n = 2; 18–24: n = 10; 25–34: n = 50; 35–44: n = 106; 45–54: n = 163; 55–64: n = 215; 65–74: n = 240; 75–84: n = 130; 85 + : 8. SSc was predominant in females (801 out of 924 cases, 86.7% of the total) with an overall sex ratio F/M of 6.5.

The prevalence, estimated at 31st December 2017, was 22.2 per 100,000 (Confidence Interval 95%, CI 95% 20.7–23.8), with significantly statistical differences among age classes (*p* < 0.0001). In particular, a decreasing prevalence was observed across age classes according to the following order: ≥ 65 years (33.2 per 100,000, CI 95% 29.6–37.3), 45–64 (31.3, CI 95% 28.1–34.8), 25–44 (17.5, CI 95% 14.8–20.5), < 25 (1.5, CI 95% 0.8–2.6). Male and female prevalence estimates were 5.9 and 37.5 per 100,000, respectively.

The overall incidence for the 2003–2017 period was 1.7 per 100,000 (0.5 and 2.8 per 100,000 for males and females, respectively). Incidence across the 5-year periods 2003–2007, 2008–2012, and 2013–2017 did not differ significantly (*p* = 0.56), ranging from 1.5 to 1.8 per 100,000.

The overall mean age at diagnosis was 59.4 ± 14.6, without significant differences (*p* = 0.61) among males and females (58.8 ± 15.1 and 59.5 ± 14.6, respectively).

### Hospitalization

The linkage between the study population and the hospital discharge database was possible for 899 out of 924 cases of SSc (97.3%); 76.5% (n = 688) of the investigated cohort had at least one admission in the study period. Hospitalization was significantly higher in males than in females (*p* < 0.01; 86.8 and 74.9%, respectively).

A total of 4111 admissions and a total LOS of 28,921 days with a mean length of inpatient stay of 7.0 days were observed, without detecting statistically significant differences between males and females (7.3 and 7.0, respectively). The average number of hospital admissions for each case was equal to 6 (6.3 for males, 5.9 for females).

Cardiovascular (40.8%) and pulmonary (36.2%) diseases constituted the majority of SSc complications (Table 1). Among these two disease groups, chronic ulcer of skin (20.4%) and lung involvement (28.1%) were the most frequent diseases.

**Table 1:** SSc-related complications and comorbidities in inpatient population in the 2003–2017 period: number of cases and percentages by sex, referred to the inpatient population and test to assess statistically significant sex differences

		Cases	%	pr-test
Heart and Circulation^a^	M	55	45.5	0.26
	F	312	40.1	
	Total	367	40.8	
Acute myocardial infarction	M	5	4.1	0.18
	F	16	2.1	
	Total	21	2.3	
Subarachnoid and intracerebral haemorrhage	M	1	0.8	0.83
	F	8	1.0	
	Total	9	1.0	
Ischemic stroke events	M	2	1.7	0.72
	F	10	1.3	
	Total	12	1.3	
Congestive heart failure	M	16	13.2	0.46
	F	85	10.9	
	Total	101	11.2	
Malignant essential hypertension	M	0	0.0	0.23
	F	9	1.2	
	Total	9	1.0	
Gangrene	M	2	1.7	0.48
	F	22	2.8	
	Total	24	2.7	
Chronic ulcer of skin (except pressure ulcer)	M	28	23.1	0.42
	F	155	19.9	
	Total	183	20.4	
**Lung**^**b**^	M	53	43.8	0.06
	F	272	35.0	
	Total	325	36.2	
PAH	M	5	4.1	0.33
	F	50	6.4	
	Total	55	6.1	
Lung involvement	M	38	31.4	0.39
	F	215	27.6	
	Total	253	28.1	
Pulmonary fibrosis	M	10	8.3	**0.04**
	F	31	4.0	
	Total	41	4.6	
Pneumonia	M	16	13.2	**0.002**
	F	44	5.7	
	Total	60	6.7	
Pulmonary embolism	M	0	0.0	–
	F	13	1.7	
	Total	13	1.4	
**Kidney**^**c**^	M	5	4.1	0.91
	F	34	4.4	
	Total	39	4.3	
Chronic kidney disease	M	1	0.83	0.48
	F	13	1.67	
	Total	14	1.56	
Acute renal failure	M	4	3.3	0.95
	F	25	3.2	
	Total	29	3.2	
**Gastrointestinal tract**^**d**^	M	5	4.1	0.56
	F	24	3.1	
	Total	29	3.2	
Hemorrhage of gastrointestinal tract	M	0	0.0	–
	F	4	0.5	
	Total	4	0.4	
Intestinal obstruction (without hernia)	M	5	4.1	0.39
	F	21	2.7	
	Total	26	2.9	
**Metabolism**^**e**^	M	43	35.5	**0.0001**
	F	153	19.7	
	Total	196	21.8	
Diabetes mellitus	M	19	15.7	**0.04**
	F	75	9.6	
	Total	94	10.5	
Gout	M	32	26.4	**0.0001**
	F	98	12.6	
	Total	130	14.5	
**Malignant neoplasms**	M	18	14.9	0.55
	F	100	12.9	
	Total	118	**13.1**	

%, percentages referred to the total of the inpatient population; pr-test, *p*-value of the test of difference of proportion, a *p*-value < 0.05 (in bold) indicates a statistically significant difference in SSc-related complications between males and females

^a^Cases with at least one occurrence of the following complications or comorbidities: acute myocardial infarction, subarachnoid and intracerebral haemorrhage, ischemic stroke events, congestive heart failure, malignant essential hypertension, gangrene, and chronic ulcer of skin

^b^Cases with at least one occurrence of the following complications or comorbidities: PAH, lung involvement in systemic sclerosis, pneumonia, and pulmonary embolism

^c^Cases with at least one occurrence of the following complications or comorbidities: chronic kidney disease and acute renal failure

^d^Cases with at least one occurrence of the following complications or comorbidities: haemorrhage of gastrointestinal tract and intestinal obstruction

^e^Cases with at least one occurrence of the following complications or comorbidities: diabetes mellitus and gout

Malignant neoplasms of bone, connective tissue, skin, and breast (30.5%), genitourinary organs (17.8%), respiratory and thoracic organs (16.9%), and digestive and peritoneum (15.3%) were the most frequent among the 118 cases of tumours observed in our study.

Males were more represented than females for respiratory diseases such as pulmonary fibrosis (8.3% vs 4.0%, *p* = 0.04) and pneumonia (13.2% vs 5.7%, *p* = 0.002), for diabetes mellitus (15.7% vs 9.6%, *p* = 0.04), and for gout (26.4% vs 12.6%, *p* = 0.0001).

Among the main causes of hospitalization, diseases of the circulatory and respiratory systems were the most frequent, with percentages of 35.2% and 22.1%, respectively, calculated on the total inpatient cases (Table 2).

**Table 2:** Causes of hospitalization among inpatient cases of SSc

Cause of hospitalization	ICD9-CM	Frequency	%
Diseases of the circulatory system	390–459	242	35.2
Diseases of the respiratory system	460–519	152	22.1
Diseases of the digestive system	520–579	124	18.0
Diseases of the skin and subcutaneous tissue	680–709	83	12.1
Malignant neoplasms	140–209, 230–239	79	11.5

Significant sex differences (*p* < 0.05) were observed for diseases of the circulatory system, with higher hospitalization among males (43.8%) than females (33.6%), and for diseases of the respiratory system, with a percentage of 26.5% for males and 21.3% for females. In particular, within the respiratory group, males resulted to be hospitalized at a significantly higher percentage than females for interstitial lung diseases (ICD9-CM: 515–517, 9.5% vs 4.6%, *p* = 0.01).

### Survival

During the study period, 132 patients died (22 males and 110 females). The average age at death was 74.0 years (range: 42–97 years); 72.4 and 74.3 years for males and females, respectively.

Overall survival rates at 1, 3, 5, and 10 years from diagnosis were 98.4%, 95.7%, 91.6%, and 79.4%, respectively.

**Fig. 1:**
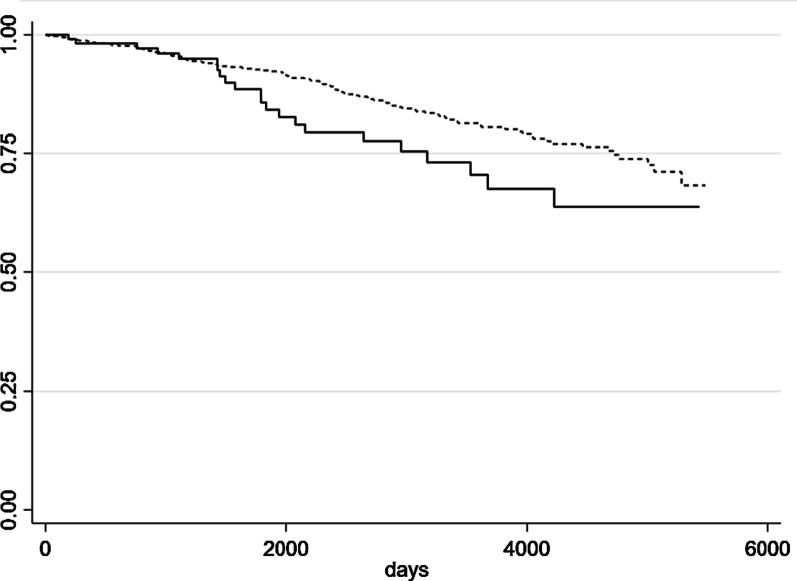
Kaplan–Meier survival curves by sex (males and females in continue and dotted lines, respectively)

The Kaplan–Meier survival analysis and the long-rank test demonstrated a higher survival in females than in males, even if borderline significant (*p* = 0.06) (Fig. 1); the survival rates at 5 year were 85.6% of males and 92.4% of females, respectively. The survival at 5 years for age classes 45–64 and 65+ years was 96.4% and 82.8%, respectively. No deaths were observed for cases under 45 years-old.

The Hazard Ratio (HR) for females was 0.65, borderline significant (CI 95% 0.41–1.03) after adjustment for age at diagnosis. HR significantly increased with age at diagnosis, resulting to be 1.09 (CI 95% 1.07–1.11) for each year increase, also after adjustment for sex.

Table 3 reported HRs related to the main investigated complications adjusted for sex and age at diagnosis.

**Table 3:** Hazard ratio between SSc-related complications and comorbidities and survival (statistically significant associations are reported in bold)

SSc-related complications and comorbidities	HR^a^	CI 95%
Acute myocardial infarction	2.14	0.93–4.89
Subarachnoid and intracerebral haemorrhage	**2.26**	1.32–3.87
Ischemic stroke events	1.30	0.24–3.63
Congestive heart failure	**3.54**	2.38–5.28
Malignant essential hypertension	3.68	0.97–14.0
Gangrene	**2.30**	1.27–4.14
Chronic ulcer of skin (except pressure ulcer)	1.36	0.95–1.95
PAH	**2.24**	1.41–3.55
Lung involvement	**1.50**	1.06–2.11
Pulmonary fibrosis	**2.60**	1.56–4.35
Pneumonia	1.59	1.00–2.53
Pulmonary embolism	1.55	0.55–4.35
Chronic kidney disease	1.30	0.58–2.88
Acute renal failure	**2.74**	1.62–4.63
Haemorrhage of gastrointestinal tract	2.69	0.73–9.93
Intestinal obstruction (without hernia)	**3.39**	2.03–5.66
Diabetes mellitus	1.31	0.84–2.04
Gout	1.35	0.94–1.95
Malignant neoplasm	**1.88**	1.27–2.80

HR, hazard ratio; CI95%, confidence interval

^a^HRs are adjusted for sex and age at diagnosis

Among the cardiovascular disorders, subarachnoid and intracerebral haemorrhage, congestive heart failure, and gangrene were associated with a lower survival. A higher risk of mortality was also associated to pulmonary fibrosis, lung involvement and PAH, among the pulmonary complications, and to acute renal failure, intestinal obstruction, and malignant neoplasms.

In order to evaluate the net effect of SSc-related complications and comorbidities on survival, a Cox regression model including all complications was developed. Congestive heart failure (HR = 2.76, CI 95% 1.80–4.25), subarachnoid and intracerebral haemorrhage (HR = 2.33, CI 95%1.21–4.48), pulmonary involvement (lung involvement and pulmonary fibrosis, and PAH) (HR = 1.66, CI 95% 1.17–2.35), and malignant neoplasms (HR = 1.63, CI 95% 1.06–2.52), were confirmed to be significantly associated to a lower survival also after adjustment for all other complications. Renal involvement (chronic kidney disease and acute renal failure) (HR = 1.66, CI 95% 0.99–2.79) and gangrene (HR = 1.73, CI 95% 0.93–3.20) resulted to be borderline significant.

### Pharmacoepidemiology

**Fig. 2:**
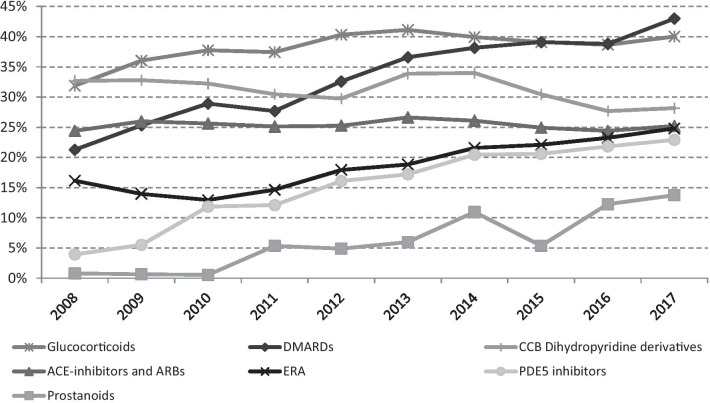
Prevalence trend (2008–2017) of the investigated drug classes. Glucocorticoids: methylprednisolone and prednisone; Disease-modifying antirheumatic drugs (DMARDs): azathioprine, cyclophosphamide, ciclosporin, methotrexate, hydroxychloroquine, mycophenolic acid, and rituximab; Calcium Channel Blockers (CCB) dihydropyridine derivatives; ACE inhibitors and ARBs (Angiotensin II Receptor Blockers) (plain and in association with dihydropyridine derivatives); Endothelin Receptor Antagonists (ERA): bosentan, ambrisentan, and macitentan; PDE-5 inhibitors (phosphodiesterase-5 inhibitors): tadalafil, vardenafil, and sildenafil; Prostanoids (prostacyclin analogues): iloprost, epoprostenol, treprostinil

Looking at the prevalence of use of the investigated drug classes, disease-modifying antirheumatic drugs—DMARDs (azathioprine, cyclophosphamide, ciclosporin, methotrexate, hydroxychloroquine, mycophenolic acid, rituximab) showed the higher percentage of users in the last years (43%), characterized by a continuous increasing trend since 2008 (Fig. 2).

Glucocorticoids (prednisone and methylprednisolone) showed an increasing prevalence from 2008 (31.9%) to 2013 (41.1%), followed by a rather steady trend in the last four years (2014–2017).

**Fig. 3:**
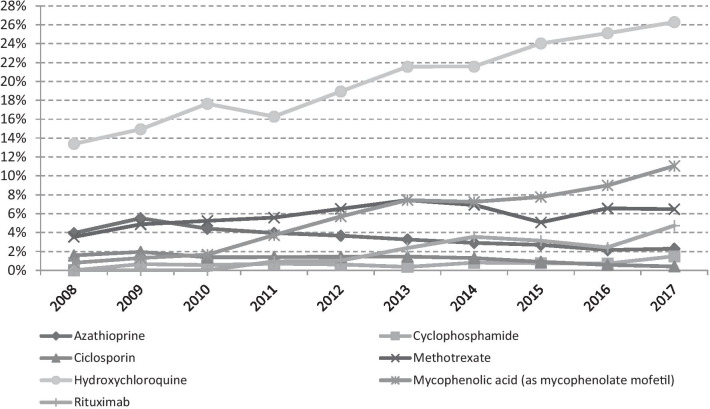
Prevalence trend (2008–2017) of the investigated disease-modifying antirheumatic drugs (DMARDs): azathioprine, cyclophosphamide, ciclosporin, methotrexate, hydroxychloroquine, mycophenolic acid (as mycophenolate mofetil), and rituximab

Among the DMARDs, hydroxychloroquine had the highest number of users: in 2017 more than 1 out of 4 cases (26.3%) had at least one prescription of this drug (Fig. 3). An increasing trend was also observed for mycophenolic acid, whose prevalence increased from 0.8 to 11.1% in the 2008–2017 period. Rituximab also showed an increasing trend, but with lower percentages of users, with a maximum of 4.7% in 2017. A steady trend of the percentage of users of methotrexate was observed from 2012 to 2017 (ranging from a minimum of 6.5% in 2012 to a maximum of 7.4% in 2013), with an increase observed from 2008 (3.5%) to 2012 (6.5%). Azathioprine and ciclosporin showed decreasing trends, the former was almost halved from 2008 (3.9%) to 2017 (2.3%), the latter decreased from 1.6% in 2008 to 0.4% in 2017. Cyclophosphamide showed a very low prevalence of use, with a maximum of 1.5% observed in 2017.

Phosphodiesterase-5 (PDE-5) inhibitors (tadalafil, vardenafil, and sildenafil) and Endothelin Receptor Antagonists (ERA) (bosentan, ambrisentan, and macitentan), both recommended for PAH and digital ulcers, had a similar trend in the 2010–2017 period. The increase was more pronounced in PDE-5 inhibitors (varying from 3.9 to 22.9%) than in ERA  (varying from 16.5 to 25.5%).

ACE inhibitors and Angiotensin II Receptor Blockers (ARB) (considered as plain and in association with dihydropyridine derivatives) showed a steady trend in the period under investigation ranging from a minimum of 24.4% (in 2008) and a maximum of 26.6% (in 2013).

The calcium channel blockers (CCB) dihydropyridine derivatives were the only ones characterized by a decreasing trend from 32.7 to 28.2%, with a higher percentage of users (34%) observed in the 2013–2014 period.

Prostanoids (prostacyclin analogues: iloprost, epoprostenol, treprostinil) showed an increasing trend from 2011 (5.3%) to 2017 (13.7%); among them, iloprost was the most used.

Looking at the intensity of use, expressed as defined daily dose (DDD) per 1000 cases per day, ACE inhibitors and ARB, DMARDs, PDE-5 inhibitors and ERA showed a steady increasing trend from 2008 to 2017 reaching a maximum of 539.2, 269.1, 160.8 and 159.0 DDD/1000 cases/day, respectively.

For CCB dihydropyridine derivatives a steady trend in use was observed along the investigated period, ranging from a minimum of 328.6 DDD/1000 cases/day in 2012 to a maximum of 377.7 in 2017.

Glucocorticoids methylprednisolone and prednisone were the only ones with a decreasing trend varying from 248.4 DDD/1000 cases/day in 2008 to 215.8 in 2017.

Among the DMARDs, hydroxychloroquine was the most used with a pronounced increasing trend from 2008 (74.8 DDD/1000 cases/day) to 2017 (150.7 DDD/1000 cases/day).

Other drugs or classes of drugs were also investigated. Proton-pump inhibitors, that should be used for the treatment of SSc-related gastro-esophageal reflux diseases (GERD) (strength of recommendation B according to EULAR) [28], show a steady trend for prevalence of use, ranging from 69 and 72%.

Riociguat, a stimulator of soluble guanylate cyclase (sGC) that has been recently approved for the treatment of PAH, showed no prescriptions before 2017.

Among the innovative therapies, in the last years the prescription of tocilizumab, an interleukin 6 inhibitor, and high dosage of polyclonal intravenous immunoglobulins (IvIg) has started in patients with SSc. Indeed, although the numbers of patients treated with tocilizumab were limited (1 case in 2014 and 3 cases each year in the 2015–2017 period), the prescription of IvIg showed a more rapid increase, ranging from 1 case in 2010 (prevalence 0.3%) up to 18 cases in 2017 (2.4% of the investigated sample).

## Discussion

The study provides a whole epidemiological profile for SSc, underlining the importance of integrating cases of a rare disease collected by a population-based registry through a multi-database approach, in order to characterize the disease in terms of survival, comorbidities and drug prescriptions.

The sex ratio, together with the observed differences of both prevalence and incidence estimates between males and females, could lead to hypothesize clinically important sex differences. Literature data suggest that the difference is even more pronounced during the reproductive period, and the increased expression of inflammatory mediators during pregnancy may play a role in this aspect [31]. However, looking at the complications, survival, and survival adjusted for risk factors, our results showed that there is a different clinical pattern according to sex in patients with SSc. Males presented a more severe disease than females resulting in a lower survival at 5 years, with evident and significant differences in respiratory complications and metabolic comorbidities. Our results suggest that males should be monitored since the early stage of the disease, in order to: (1) detect systemic involvement even in the absence of other clinical symptoms; (2) identify the best therapeutic strategy to prevent negative clinical outcomes and improve the prognosis.

The prevalence calculated in our study (22.2 per 100,000) was in agreement with estimates observed by a recent systematic review on SSc by Bergamasco et al. reporting highly heterogeneous prevalence estimates across 11 studies performed in Europe, ranging from 7.2 to 33.9 per 100,000 individuals [32]. Discrepancies among studies of SSc prevalence are mainly due to different case classification and different methods of retrieval of cases (registries, hospitals, or general practitioners, individually or in multi-source approach).

The incidence of 1.7 per 100,000 for the 2003–2017 period observed in our study, with a difference between males and females (0.5 and 2.8 per 100,000, respectively), was consistent with the estimates of annual incidence of five European studies (ranging from 0.6 to 2.3), who also reported a higher incidence in females than in males (1.8 vs 0.7 per 100,000) [32].

The study population, with an observed overall mean age at diagnosis of 59.4, appeared to be similar to those reported by Bergamasco et al., ranging from 50.2 to 59.8 (only age at diagnosis considered). We have not observed significant differences in age at diagnosis between males and females, in contrast with Alamanos et al. who found women to have a lower mean age compared to men (49.2 ± 15.7 and 58.9 ± 13.5 years, respectively) [7].

### Hospitalization

Concerning the cardiovascular complications and comorbidities, proportion observed in our study for myocardial infarction (2.3%) and ischemic stroke events (1.3%) are consistent with what reported by Butt et al. (3% for both comorbidities) [18], Man et al. (2.3% for myocardial infarction and 2.5% for ischemic stroke events) [10], and Chu et al. (2.3% for myocardial infarction) [33]. Our estimates are slightly lower respect to what reported by Aviña-Zubieta et al. (4.7% and 2.9%) [34], but in this case, the Authors declared that their study showed a higher risk of both myocardial infarction and stroke in comparison to previous studies, maybe because they utilized both hospitalization data and death certificate codes. Percentage of congestive heart failure observed in our study (11.2%) are in the wide range of values observed by Butt et al. (3%) [18] and Amoda et al. (24.4%) [17]. This heterogeneity mainly depends on different definitions of both SSc cases and heart failure.

We observed 2.7% of cases with gangrene which is not directly comparable to a few studies reporting 1.7%, 3.9% and 5% of cases with peripheral artery disease [10, 18, 33]. However, the sum of cases of chronic ulcer of skin (20.4%) and gangrene (2.7%) observed in our cohort is consistent with the findings of Noviani et al. who reported 24.3% of cases with digital ulcers or gangrene [15].

Concerning the pulmonary complications, we observed a prevalence of PAH of 6.1%, which is consistent with literature data, reporting heterogeneous estimates ranging between 4.9 and 26.7% depending on the applied diagnostic tools and definitions [35].

We found 31.2% of total pulmonary involvement (28.1% of cases with lung involvement and 3.1% with pulmonary fibrosis as defined with ICD-9-CM). Our results are not directly comparable to other studies, as we defined lung involvement according to ICD-9-CM codes and we do not have data on the prevalence of pulmonary fibrosis alone based on high-resolution computerized tomography of the lungs.

Concerning the renal complications, our prevalence of 4.3% was slightly higher than the proportion reported in a hospital-based study developed in Northwestern Spain (1% of SSc-related renal crisis) [9]. This difference could be explained by the inclusion of non-SSc-related renal failure in our prevalence estimate of 4.3%, since in our database we were not able to identify specific SSc-related renal failure.

Concerning the metabolic comorbidities, percentage of diabetes mellitus among our inpatient population, (10.5%) was consistent with what observed by Amoda et al. (11.4% for the sum of diabetes with and without other associated complications) [17] and by Chu et al. (12.1%) [33].

Concerning the malignant neoplasms, the observed percentage of 13.1% was slightly higher but consistent with what reported in literature. In particular, Pagkopoulou et al. reported in a recent review that malignancies in patients with SSc occur in a percentage of 3.6–10.7%, but with varying incidence estimates among studies reflecting the heterogeneity of SSc as well as the epidemiologic variations of the disease across different countries [36].

### Survival and risk factors

Our results for survival at 5 and 10 years (91.6% and 79.4%, respectively) were higher than those reported by Bergamasco et al., who identified three publications reporting survival rates in the ranges of 83–84% and 65–73% at 5 and 10 years, respectively [7, 9, 32, 37]. This discrepancy can have a reason in the fact that these studies have been developed on data referred to preceding decades (1988–2006, 1981–2002, and 1983–2005) and suggests that the survival of SSc patients may have improved over the last decade. This hypothesis is plausible, taking into account the improvement occurred in the last years in terms of early diagnosis and consequently of treatment, not only of SSc as a whole, but also of its complications and comorbidities that may have a negative impact on patients’ survival.

Confirming results come from Butt et al. who recently investigated the mortality-rates of SSc in the 1995–2015 period, observing the 1-year all-cause mortality rate per 100 person-years decreasing from 6.1 in 1995 to 5.3 in 2015 [18].

Regarding the effect of SSc-related complications and comorbidities on survival, our results were consistent with what was recently reported in a meta-analysis by Pokeerbux et al., who observed that age at diagnosis, male sex, renal involvement and SSc-related renal crisis, interstitial lung disease, cardiac involvement, and cancer were significantly associated with a worse prognosis [16].

In our study, both congestive heart failure and renal involvement were associated to poor prognosis (lower survival). More than half of the cases (20 out of 39) with renal involvement (chronic kidney disease or acute renal failure) also presented congestive heart failure, thus suggesting that heart failure may cause renal failure, probably due to the cortical renal hypoperfusion, as hypothesized by other Authors [38].

We observed that intestinal occlusion was associated to a lower survival. Very few studies investigated this association; Mecoli et al. evidenced a higher mortality in SSc patients with acute intestinal pseudo-obstruction who had low haemoglobin and serum albumin levels at presentation [39].

### Pharmacoepidemiology

The analysis on drug prescriptions allows identifying an increase of use for different categories of drugs. Being SSc a complex disease, the therapeutic approach includes several immuno-active and vasoactive drugs.

Concerning the immune-active drugs, glucocorticoids, although associated with a higher risk of scleroderma renal crisis [28], are frequently prescribed as they are part of the therapeutic strategy in the management of inflammatory arthritis. Recent evidences suggest that using glucocorticoids at a low-dosage is relatively safe [40] and may be helpful in patients with SSc, in particular in those with an early disease characterized by a prevalent inflammation [41]. This is in line with what observed in our study since methylprednisolone and prednisone were the only drugs with a decreasing trend in term of intensity of use along the 2008–2017 period. As reported in literature, a long-term use or high doses of glucocorticoids is associated with numerous side effects such as hyperglycaemia or diabetes [42]. Despite this, we did not observe in our cohort an increase of diabetes following the therapy with glucocorticoids, probably due to the low dosage usually prescribed in SSc. In particular, we observed 43 cases with diabetes out of 584 cases treated with glucocorticoids (6.9%) and 9 cases with diabetes out of 207 cases not treated with glucocorticoids (4.2%). Despite the higher percentage, the difference between these proportions was not statistically significant (*p* = 0.156).

As glucocorticoid use may be insufficient and burdened by adverse events, the use of DMARDs is often proposed. Immunosuppressants may allow a better disease control and their use is also recommended in the most recent EULAR guidelines for treatment of SSc [28]. In our cohort, the results confirmed a significant increased prevalence and intensity of use of immunosuppressants during the study period. In particular, there was an increase of the use of mycophenolate mofetil and cyclophosphamide (even if with a lower prevalence) and this is probably due to the recognized effectiveness of these two treatments for interstitial lung disease, pulmonary fibrosis, skin and cardiac involvement [43, 44]. On the contrary, the prevalence of use of azathioprine and cyclosporine-A decreased, in the latter case it is probably due the possible association with the scleroderma renal crisis [45].

Methotrexate has been recommended by EULAR (strength A) for the treatment of skin manifestations of early diffuse SSc [28] and, similarly to what reported by Panoupulos et al. [46], it has been the most prescribed immunosuppressant for this condition until 2014, when it was overcome by mycophenolate mofetil, as also observed in our study.

Hydroxychloroquine is the most used DMARD and the percentage of treated patients showed a constant increasing trend, likely for beneficial impact on joint involvement, as recently observed [47].

The use of rituximab, a B-cell depleting monoclonal antibody, increased in the last years for the treatment of skin and lung involvement, although recently published data are contrasting [48, 49].

We also observed in the last years an increase in prescriptions of tocilizumab and intravenous globulins, representing an innovative vanguard approach for the treatment of refractory patients [50, 51].

The prevalence of use of vasoactive drugs, such as prostanoids, ERA and PDE-5 inhibitors, progressively increased along the study period. On the other hand, the number of patients treated with CCB dihydropyridine derivatives decreased during the time, even if CCB was the class of vasoactive drugs with the highest observed prevalence of use in our study, confirming data recently published by Blagojevic et al. in a European multicentre study [52]. The gradual decreasing trend in prevalence of use of CCB, may be explained with the necessity to avoid hypotensive attacks in patients already treated with ERA and PDE-5 inhibitors.

The overall prevalence of use of prostanoids, recommended for PAH, digital ulcers and Raynaud’s phenomenon, could be underestimated because before 2011 prostanoids were prescribed to inpatient cases only and the drug prescription database available for this study collects information only referred to outpatient prescriptions (see Methods section).

In general, this study demonstrates the importance of integrating data collected by a population-based registry with information from health administrative databases in defining a complete epidemiological profile of SSc. In particular, using a multi-database approach allows taking advantage to the greatest possible extent of the capacity of a population-based registry to collect almost all the cases diagnosed in a defined geographical area and, at the same time, exploiting information about comorbidities, therapies and vital status, routinely collected at local level.

This study also presents limitations. Firstly, some SSc-related complications could not be well defined, as the information derived from an administrative database (hospital discharge database) contains a lower level of details than disease-specific clinical data usually collected in hospital-based databases (e.g. scleroderma renal crisis is not defined by itself). Secondly, information about drug utilization do not take into account inpatient prescriptions (neither day-hospital prescriptions, except in the last years). This could lead to an underestimation of use for some drugs such as prostanoids, as described before. Another limitation could be that the diagnoses were made taking into account the ARA criteria until 2013 and EULAR/ACR from 2013 to 2017. This could lead to some difference in the classification of patients, although we did not observe a significant increased incidence after the introduction of the EULAR/ACR criteria.

## Conclusion

The study provides a complete epidemiological characterization of SSc, in terms of prevalence and incidence, survival, disease-related complications and comorbidities and therapies, by using a multi-database population-based approach. Prevalence estimate was 22.2 per 100,000, with the highest prevalence observed for the age class over 65 years old (33.2 per 100,000, CI 95% 29.6–37.3). SSc was found to be predominant in females (86.7% on the total) with an overall sex ratio F/M of 6.5 and an incidence of 2.8 and 0.5 per 100,000 for females and males, respectively. Despite this, males presented a more severe disease than females corresponding to a lower survival, with observed significant differences in pulmonary complications and metabolic comorbidities. DMARDs, ERA, and PDE-5 inhibitors were the drugs with the more pronounced increasing trend of prevalence of use in the 2008–2017 period.

The multi-database approach here presented is important in the field of rare diseases where it is often difficult to provide accurate epidemiological indicators, especially when cases are collected from hospital-based disease registries not covering a specific geographical area. A population-based registry can overcome this limit and, when exploited in synergy with administrative health databases, can be used to provide evidence related to disease outcomes and therapies and to assess the burden of the disease, relying on a significant cohort of cases.

Building an archive of data from multiple databases linking a cohort of patients to their comorbidities, clinical outcomes and survival, is important for the clinical management. The identification of complications or comorbidities is particularly relevant if the complication is associated with a negative clinical outcome or with a lower survival and, in this sense, the choice of an appropriate treatment for a specific complication or comorbidity can result in a better outcome.

## Supplementary Information

**Additional file 1.** Definition of diseases associated with SSc (Table 1S) and investigated drugs (Table 2S).

## Data Availability

The data that support the findings of this study are available from Regione Toscana but restrictions apply to the availability of these data, which were used under license for the current study, and so are not publicly available. Data are however available from the authors upon reasonable request and with permission of Regione Toscana.

## Abbreviations

ARBs: Angiotensin II receptor blockers
ATC: Anatomical therapeutic chemical
CCB: Calcium channel blockers
CI 95%: Confidence interval 95%
DDD: Defined daily dose
DMARDs: Disease-modifying antirheumatic drugs
ERA: Endothelin receptor antagonists
HR: Hazard ratio
ICD: International classification of diseases
IvIG: Polyclonal intravenous immunoglobulins
LOS: Length of stay
PAH: Pulmonary arterial hypertension
PDE-5: Phosphodiesterase-5
SSc: Systemic sclerosis
