# Supplementary material for: Epidemiology of systemic sclerosis: a multi-database population-based study in Tuscany (Italy)
Source: Orphanet J Rare Dis. 2021 Feb 17;16:90. doi: 10.1186/s13023-021-01733-4 (PMC7890847; doi:10.1186/s13023-021-01733-4)
Supplement: Supplementary file 1 — Additional file 1. Definition of diseases associated with SSc (Table 1S) and investigated drugs (Table 2S). [file 13023_2021_1733_MOESM1_ESM.docx]

**Additional File 1**

**Table S1.** Definition of diseases associated with SSc according to ICD9-CM codes

| **Associated disease** | **ICD-9-CM** |
| --- | --- |
| *Heart and circulation* |  |
| Acute myocardial infarction | 4100, 41000, 41001, 41002, 4101, 41010, 41011, 41012, 4102, 41020, 41021, 41022, 4103, 41030, 41031, 41032, 4104, 41040, 41041, 41042, 4105, 41050, 41051, 41052, 4106, 41060, 41061, 41062, 4107, 41070, 41071, 41072, 4108, 41080, 41081, 41082, 4109, 41090, 41091, 41092 |
| Congestive heart failure | 398.91, 40201, 40211, 40291, 40401, 40403, 40411, 40413, 40491, 40493, 4280, 4281, 42820, 42821, 42822, 42823, 42830, 42831, 42832, 42833, 42840, 42841, 42842, 42843, 4289 |
| Subarachnoid and intracerebral hemorrhage | 430, 431 |
| Ischemic stroke events | 43301, 43311, 43321, 43331, 43381, 43391, 4340, 43400, 43401, 4341, 43410, 43411, 4349, 43490, 43491, 436 |
| Malignant essential hypertension | 4010 |
| Gangrene | 44024, 7854 |
| Chronic ulcer of skin (except pressure ulcer) | 7071, 70710, 70711, 70712, 70713, 70714, 70715, 70719, 70720, 70721, 70722, 70723, 70724, 70725, 7078, 7079 |
| *Lung* |  |
| PAH | 4160 |
| Lung involvement (in systemic sclerosis) | 5172 |
| Pulmonary fibrosis | 5163, 5168 |
| Pneumonia | 00322, 0203, 0204, 0205, 0212, 0221, 0310, 0391, 0521, 0551, 0730, 0830, 1124, 1140, 1144, 1145, 11505, 11515, 11595, 1304, 1363, 4800, 4801, 4802, 4803, 4808, 4809, 481, 4820, 4821, 4822, 4823, 48230, 48231, 48232, 48239, 4824, 48240, 48241, 48242, 48249, 4828, 48281, 48282, 48283, 48284, 48289, 4829, 483, 4830, 4831, 4838, 4841, 4843, 4845, 4846, 4847, 4848, 485, 486, 5130, 5171 |
| Pulmonary embolism | 41511, 41519 |
| *Kidney* |  |
| Acute renal failure | 584, 5845, 5846, 5847, 5848, 5849 |
| Chronic kidney disease | 585* |
| *Gastrointestinal tract* |  |
| Hemorrhage of gastrointestinal tract | 5789 |
| Intestinal obstruction (without hernia) | 5600, 5601, 5602, 56030, 56031, 56032, 56039, 56081, 56089, 5609 |
| *Metabolism* |  |
| Diabetes mellitus^a^ | 25000, 25001, 25002, 25003, 25010, 25011, 25012, 25013, 25020, 25021, 25022, 25023, 25030, 25031, 25032, 25033, 25040, 25041, 25042, 25043, 25050, 25051, 25052, 25053, 25060, 25061, 25062, 25063, 25070, 25071, 25072, 25073, 25080, 25081, 25082, 25083, 25090, 25091, 25092, 25093, V4585, V5391, V6546 |
| Gout | 274, 2740, 2741, 27410, 27411, 27419, 2748, 27481, 27482, 27489, 2749 |
| *Malignant neoplasms* | 140-209, 230-239 |
| ^a^: defined in combination with information on the use of “Drugs used in diabetes” (ATC codes: A10*) | |

**Table S2.** Investigated drugs with ATC codes (Anatomical Therapeutic Chemical classification system) and recommendations of use (prevention and active treatment).

| **Drug or drug class** | **ATC code** | **Recommendation of use** |
| --- | --- | --- |
| DMARDs:  *azathioprine*  *cyclophosphamide*  *ciclosporin*  *methotrexate*  *hydroxychloroquine*  *mycophenolic acid*  *rituximab* | L04AX01  L01AA01  L04AD01  L01BA01,L04AX03  L04AA06  L01XC02  P01BA02 | SSc-ILD  SSc-ILD  SSc-related skin involvement  SSc-related cardiac involvement  SSc-related inflammatory arthritis  SSc-related skin involvement  SSc-related cardiac involvement  SSc-related cardiac involvement  SSc-related skin involvement  SSc-ILD  SSc-related cardiac involvement  SSc-ILD  SSc-related skin involvement |
| Glucocorticoids:  *methylprednisolone*  *prednisone* | H02AB04  H02AB07 | SSc-related inflammatory arthritis |
| ACE-inhibitors and ARBs^a^ | C09A*, C09BB*,  C09C*, C09DB* | SSc-RC, SSc-RP |
| CCB (dihydropiridine derivatives) | C08CA* | SSc-RCs, SSc-RP, SSc-related digital ulcers |
| ERA:  *Bosentan*  *Ambrisentan*  *Macitentan* | C02KX01  C02KX02  C02KX04 | SSc-PAH  SSc-related digital ulcers |
| Riociguat |  | SSc-PAH |
| PDE-5 inhibitors:  *Tadalafil*  *Vardenafil*  *Sildenafil* | G04BE03  G04BE08  G04BE09 | SSc-RP, SSc-related digital ulcers  SSc-PAH  SSc-PAH |
| Prostanoids (prostacyclin analogues):  *Iloprost*  *Epoprostenol*  *Treprostinil* | B01AC11 | SSc-PAH, SSc-related digital ulcers  SSc-RP  SSc-related digital ulcers |
| ^a^Both plain and in combination with dihydropyridine derivatives.  Abbreviations: DMARDs, Disease modifying antirheumatic drugs; ARBs, Angiotensin II Receptor Blockers; CCB, Calcium Channel Blockers; ERA, Endothelin Receptor Antagonists; PDE-5, Phosphodiesterase 5; SSc-ILD, Systemic Sclerosis-related interstitial lung disease; SSc-RC, SSc-related renal crisis; SSc-RP, SSc-related Raynaud’s phenomenon; SSc-PAH, SSc-related pulmonary arterial hypertension; SSc-GERD, SSc-related gastro-oesophageal reflux disease. | | |
